# Humans from *Wuchereria bancrofti* endemic area elicit substantial immune response to proteins of the filarial parasite *Brugia malayi* and its endosymbiont *Wolbachia*

**DOI:** 10.1186/s13071-016-1963-x

**Published:** 2017-01-24

**Authors:** Ruchi Jha, Mamta Gangwar, Dhanvantri Chahar, Anand Setty Balakrishnan, Mahendra Pal Singh Negi, Shailja Misra-Bhattacharya

**Affiliations:** 1 Division of Parasitology, CSIR-Central Drug Research Institute, BS 10/1, Sector 10 Jankipuram Extension, Sitapur Road, Lucknow, UP 226031 India; 2 Biometry and Statistics Division, CSIR-Central Drug Research Institute, BS 10/1, Sector 10 Jankipuram Extension, Sitapur Road, Lucknow, UP 226031 India; 3 Department of Genetic Engineering, School of Biotechnology, Madurai Kamraj University, Palkalai Nagar, Madurai, TN 625021 India; 4 Academy of Scientific and Innovative Research, New Delhi, India

**Keywords:** Lymphatic filariasis, *Brugia malayi*, *Wolbachia*, Isotype, Vaccine

## Abstract

**Background:**

In the past, immune responses to several *Brugia malayi* immunodominant antigens have been characterized in filaria-infected populations; however, little is known regarding *Wolbachia* proteins. We earlier cloned and characterized few *B. malayi* (trehalose-6-phosphate phosphatase, Bm-TPP and heavy chain myosin, BmAF-Myo) and *Wolbachia* (translation initiation factor-1, Wol Tl IF-1 and NAD^+^-dependent DNA ligase, *w*Bm-LigA) proteins and investigated the immune responses, which they triggered in animal models. The current study emphasizes on immunological characteristics of these proteins in three major categories of filarial endemic zones: endemic normal (EN, asymptomatic, amicrofilaraemic; putatively immune), microfilariae carriers (MF, asymptomatic but microfilaraemic), and chronic filarial patients (CP, symptomatic and mostly amicrofilaraemic).

**Methods:**

Immunoblotting and ELISA were carried out to measure IgG and isotype antibodies against these recombinant proteins in various clinical categories. Involvement of serum antibodies in infective larvae killing was assessed by antibody-dependent cellular adhesion and cytotoxicity assay. Cellular immune response was investigated by in vitro proliferation of peripheral blood mononuclear cells (PBMCs) and reactive oxygen species (ROS) generation in these cells after stimulation.

**Results:**

Immune responses of EN and CP displayed almost similar level of IgG to Wol Tl IF-1 while other three proteins had higher serum IgG in EN individuals only. Specific IgA, IgG1, IgG3 and IgM to Bm-TPP were high in EN subjects, while BmAF-Myo additionally showed elevated IgG2. Enhanced IgA and IgG3 were detected in both EN and CP individuals in response to Wol Tl IF-1 antigen, but IgG1 and IgM were high only in EN individuals. *w*Bm-LigA and BmAF-Myo exhibited almost similar pattern of antibody responses. PBMC isolated from EN subjects exhibited higher proliferation and ROS generation when stimulated with all three proteins except for Wol Tl IF-1.

**Conclusions:**

Overall, these findings display high immunogenicity of all four proteins in human subjects and revealed that the EN population was exposed to both *B. malayi* and *Wolbachia* proteins simultaneously. In addition, immune responses to Wol Tl IF-1 suggest possible role of this factor in *Wolbachia*-induced pathological responses while immune responses to other three proteins suggest that these can be explored further as vaccine candidates.

**Electronic supplementary material:**

The online version of this article (doi:10.1186/s13071-016-1963-x) contains supplementary material, which is available to authorized users.

## Background

Human lymphatic filariasis (LF) is a debilitating disease caused by *Wuchereria bancrofti*, *Brugia malayi* and *Brugia timori* and transmitted through mosquitoes*. Wuchereria bancrofti*, the most prevalent species worldwide, is responsible for about 80% of the infection in the endemic areas, while *B. malayi* and *B. timori* are less prevalent [1]. Approximately 120 million people are infected and 1.39 billion are at the risk of infection in tropical and subtropical areas across the globe, causing serious socioeconomic consequences [2, 3]. Mass drug administration of albendazole in conjunction with diethylcarbamazine or ivermectin is recommended for controlling LF [4]. However, these strategies have limitations associated with repeated administration of conventional drugs due to limited adulticidal activity and reports of development of drug resistance. Anti-wolbachial targeting with antibiotics against mutualistic endosymbiont *Wolbachia* has been found effective [5–8]. However, because antibiotics require several weeks treatment for macrofilaricidal activity, they are not suitable for mass administration. Discovery of new macrofilaricidal drug or a potent vaccine would be an appropriate complementary approach to control human bancroftian filariasis.

Due to complex life-cycle of parasite, involving many stages and varying host immune responses, human LF presents wide clinical spectrum. In endemic zones, the population can be grouped on the basis of clinical and parasitological status into three major categories: endemic normal (EN, asymptomatic, amicrofilaraemic completely free from any type of filarial infection; putatively immune); microfilariae carriers (MF, asymptomatic but microfilaraemic); and chronic filarial patients (CP, symptomatic and mostly amicrofilaraemic). Immune status of EN, MF and CP categories can highlight the significance of filarial antigens in protective immunity, diagnosis and/or pathogenesis. Endemic normal individuals, despite of being continually exposed to infective mosquito bites, remain immune to infection; this category suggests that an antifilarial vaccine may be feasible [9]. In recent years, rapid progress in filarial research has provided new insights into host-parasite relationship and associated immune responses, leading to the discovery of antifilarial agents and potential vaccine molecules. Many essential proteins of *B. malayi* and *Wolbachia* have been characterized in search of potential vaccine candidate and/or effective drug targets [10–16].

Heavy chain myosin of adult female *B. malayi* (BmAF-Myo), an important body wall muscle protein, and trehalose-6-phosphate phosphatase (TPP), a vital enzyme of trehalose biosynthetic pathway of filarial nematodes, serve many important physiological functions in several helminth parasites [17–21]. Both have been cloned and characterized by us [22, 23]. *Brugia malayi* TPP (Bm-TPP) and BmAF-Myo both show cross-reactivity with bancroftian human sera and provide significant protection against infective larval challenge in experimental rodent models [24–27]. *Wolbachia* translation initiation factor-1 (Wol Tl IF-1) is an excretory-secretory protein that also elicits protective immunity in rodent model [28]. We have also characterized and reported on NAD^+^-dependent DNA ligase (*w*Bm-LigA), another vital enzyme of *Wolbachia* with an important role in DNA replication, transcription and repair [29, 30].

The vital roles played by the above four proteins in filarial biology and their strong reactivity with pooled human sera collected from subjects in a bancroftian endemic area, especially those in the EN group, provided a foundation for the current investigation, which explored the sero-reactivity and cellular immune response of humans in a filaria endemic area to these recombinant proteins.

## Methods

### Parasites

Sub-periodic *B. malayi* was experimentally maintained in rodent host *Mastomys coucha* (GRA ‘Giessen’ strain) through laboratory-bred mosquito vector *Aedes aegypti*. The mosquitoes were fed on infected donor animals. On day 9 ± 1, infective larvae (L3) of *B. malayi* were recovered from fed mosquitoes by the Baermann method [15].

### Study population

Blood samples were collected at Tiruvallur district and its surrounding villages, Chennai, India. All bancroftian sera samples were collected in the same manner and time frame in March 2015. Sampling was carried out with the help of paramedical staff provided by the Primary Health Center (PHC) and collected blood samples were divided into EN, MF and CP categories. The individuals were categorized as EN, MF and CP groups based on record available at these centers as well as, physical examination, presence of microfilariae (mf) and circulating filaria antigen (CFA). Individuals who were symptom-free, negative for CFA in ICT card test and mf in thick night blood smears were grouped as EN. The inhabitants found positive in ICT and had mf in night blood smears were grouped under MF category while those displaying symptoms of clinical filarial disease such as, lymphedema, lymphadenitis, lymphangitis or elephantiasis were designated as CP category. Details of all individuals participating in the current study were listed in Additional file 1: Table S1. NEN sera samples of non-endemic zone of India (Jammu & Kashmir) collected and stored earlier at -80 °C were used.

### Overexpression and purification of *B. malayi* recombinant proteins

Genes encoding Bm-TPP and BmAF-Myo were cloned and proteins were expressed and purified as described earlier [22, 23]. In brief, Bm-TPP coding sequence (GenBank: XM_001893174) and BmAF-Myo coding sequence (GenBank: AY705730) were PCR amplified from cDNA of adult worms, cloned into topo T/A (3.9 bp) vector and subcloned in expression vector pET28a (+) and pET28b, respectively. After transformation of the recombinant constructs in competent *Escherichia coli* (DE3) BL21 (Novagen, Madison, WI), logarithmic phase culture was induced with 0.5 mM isopropyl β-D thiogalactoside (IPTG; Thermo Scientific, Waltham, Massachusetts, United States) for 5 h at 37 °C (Bm-TPP) and 30 °C (BmAF-Myo) at 220 rpm for protein overexpression. The cells were harvested, and the pellet resuspended in 50 mM sodium phosphate buffer (pH 8.0) containing 300 mM NaCl, 2 mM phenylmethylsulfonyl fluoride (PMSF) and 10 mM imidazole. The cells were disrupted in a sonicator and centrifuged, and proteins were purified by nickel nitrilotriacetic acid (Ni-NTA; Qiagen, Hilden, Germany) agarose affinity column. The purified proteins were dialyzed overnight against 50 mM NaH_2_PO_4_ at 4 °C and protein concentration was determined by Bradford method [31]. Single band of proteins were obtained on 10% sodium dodecyl sulfate-polyacrylamide gel electrophoresis (SDS-PAGE), confirmed the purity of protein in eluted fraction. Both the recombinant proteins had < 1 EU/mg LPS contamination as determined by toxin sensor limulus amebocyte lysate (LAL) assay kit (Thermo Fisher Scientific, Waltham, Massachusetts, United States) which was removed by polymyxin B agarose resins column (Sigma-Aldrich, St. Louis, Missouri, USA).

### Overexpression and purification of *Wolbachia* recombinant proteins

We earlier reported on the cloning of genes encoding Wol Tl IF-1 and *w*Bm-LigA of *B. malayi* endosymbiont *Wolbachia* and expression and purification of recombinant proteins [28, 29]. Briefly, *wol infA* (NCBI 3266784) and *wBm-ligA* genes (2,052 bp) encoding Wol Tl IF-1 and *w*Bm-LigA were PCR-amplified, cloned into pTZ57R/T (2.88 kb) vector, subcloned into pET28a(+) vector (Novagen, Madison, WI) and transformed into Rosetta cells. Recombinant proteins were over-expressed by 0.5 mM IPTG for 6 h at 25 °C. The cell pellet was suspended in Buffer A (20 mM Tris-Cl pH 8.0, 250 mM NaCl, 10 mM imidazole) in presence of lysozyme (1 mg/ml) and Triton X-100 (0.1%) for cell lysis for *w*Bm-LigA. The cell pellet for Wol Tl IF-1 protein was suspended in 50 mM NaH_2_PO_4_ buffer (pH 8.0) containing 250 mM NaCl, 1 mM PMSF and 10 mM imidazole. These proteins were purified by Ni-NTA column and analyzed on 10% SDS-PAGE as single band. The protein content estimation, endotoxin level determination and removal were done as described above.

### Screening of serum antibodies to Bm-TPP, BmAF-Myo, Wol Tl IF-1 and *w*Bm-LigA by immunoblotting

Sero-reactivity of human bancroftian subjects with all the four recombinant proteins was observed in Western blot using sera of different categories (EN, *n* = 24; MF, *n* = 21; CP, *n* = 24; NEN, *n* = 10) as primary antibody. Purified recombinant proteins along with prestained molecular weight protein marker (Puregene, Genetix, New Delhi, India) were run on 10% or 15% (Wol Tl IF-1) SDS-PAGE and transferred to polyvinylidene fluoride (PVDF) membrane (TE 77 PWR Semi-dry transfer unit, Amersham Biosciences, Little Chalfont, United Kingdom). Membrane was cut into strips, blocked in 3% skim milk for 2 h at room temperature, washed thrice with PBS containing 0.5% Tween 20 (PBST), and individually incubated at 25 °C with individual patient serum sample of different categories at 1:800 dilution. Strips were re-washed with PBST and re-incubated with anti-human IgG horseradish peroxidase conjugate (Sigma-Aldrich, St. Louis, Missouri, USA) at 1:10,000 dilution for 2 h at 25 °C. The strips were later developed with 3,3′-diaminobenzidine tetra hydrochloride (DAB) in presence of H_2_O_2_ (Sigma-Aldrich, St. Louis, Missouri, USA).

### Enzyme-linked immunosorbent assay (ELISA)

Serum antibody levels to each protein in each group of patients were determined by indirect ELISA as described previously [25]. In brief, ELISA plates (Nunc, Roskilde, Denmark) were coated with 1 μg/ml each of Bm-TPP, BmAF-Myo, Wol Tl IF-1 and *w*Bm-LigA (100 μl/well) in carbonate buffer (pH 9.6) separately and incubated overnight at 4 °C. After three washings with PBST, plates were blocked with 200 μl/well of 3.5% skim milk for 2 h at 37 °C. Pooled human sera of each category were used as primary antibodies and antibody titers were obtained by 2-fold serial dilutions (1:50 to 1:25,600) in triplicate. Primary antibody (100 μl/well) was added and plates were incubated at 37 °C for 2 h, followed by washing and re-incubation at 37 °C for 1 h in presence of anti-human IgG-HRP (1:10,000). The reaction was developed by orthophenylenediamine (OPD) and terminated by adding 2.5 N H_2_SO_4_. The absorbance was read at 492 nm in ELISA multi-plate reader (Tecan, Schweiz, AG, Switzerland). All individual sera (EN, *n* = 20; MF, *n* = 20; CP, *n* = 20 and NEN, *n* = 10) were subsequently tested in ELISA at 1:800 dilution and IgG antibody level was determined in individual serum samples of each category after coating the plates with each recombinant protein.

### Measurement of antibody isotypes

Different isotypes were also measured in the sera of each patient of all categories. Briefly, ELISA plates were coated with 0.1 μg/ml of each protein for overnight at 4 °C, blocked, washed and re-incubated with primary antibody (human sera of different groups) at 1:800 dilution. After washing, ELISA plates were re-incubated with HRP conjugated anti-human IgA, IgM, IgE, IgG1, IgG2, IgG3 and IgG4 monoclonal antibodies (Abcam, Cambridge, UK) as secondary antibodies (1:5,000). Color was developed as above and absorbance was recorded at 492 nm using an ELISA reader.

### Isolation of human peripheral blood mononuclear cells (PBMCs)

Heparinized venous blood of EN, MF and CP (5 patients/category) were collected and diluted 1:1 with PBS. PBMCs were isolated by layering diluted blood on Histopaque 10771 (Sigma-Aldrich, St. Louis, Missouri, USA) and tubes were centrifuged at 400× *g* for 30 min. Cells were collected from the interface of histopaque and plasma, washed twice with Roswell Park Memorial Institute medium 1640 (RPMI 1640) containing 1% antibiotic-antimycotic solution (Cell clone, Genetix, New Delhi, India), re-centrifuged and finally suspended in complete RPMI (C-RPMI 1640), fortified with 1% antibiotic-antimycotic solution and 10% FBS (Sigma-Aldrich, St. Louis, Missouri, USA) [32]. Trypan blue dye exclusion method was used to check the viability of cells.

### Depletion of bancroftian serum antibodies to Bm-TPP, BmAF-Myo, Wol Tl IF-1 and *w*Bm-LigA

Anti-Bm-TPP, anti-BmAF-Myo, anti-Wol Tl IF-1 and anti-*w*Bm-LigA antibodies present in the pooled sera of EN, MF and CP individuals (detected in blot and ELISA) were depleted by repeated binding of sera with Bm-TPP, BmAF-Myo, Wol Tl IF-1 and *w*Bm-LigA coupled Ni-NTA agarose resins as described previously [26]. In brief, 1 mg of his-tagged recombinant proteins were coupled to Ni-NTA resins individually at 4 °C overnight. Resins were washed with PBS to remove unbound proteins and incubated with 200 μl of pooled sera at 4 °C. After centrifugation, supernatant was collected. This antibody binding was repeated until supernatant did not react with recombinant protein in ELISA. The antibody-depleted sera were later used in antibody-dependent cell-mediated cytotoxicity (ADCC) assay.

### Antibody-dependent cell-mediated cytotoxicity (ADCC) assay

In vitro ADCC assay was performed as described previously [14, 33, 34]. Briefly, in a 96-well culture plate (Becton Dickinson, Franklin Lakes, New Jersey,USA), approximately 10–20 L3 of *B. malayi* were cultured in triplicates with 0.2 × 10^6^ PBMCs (collected from a normal healthy human volunteer) and 50 μl each of pooled EN, MF, CP and NEN sera or protein-depleted EN, MF and CP sera. Sera of each group were pooled by taking equal quantity from all 20 individuals of the group. Each well contained 200 μl RPMI 1640 media and plate was incubated for 48 h at 37 °C in presence of 5% CO_2_. The experiment was repeated thrice. The larval viability was determined microscopically; viable larvae moved actively while dead larvae were flaccid, damaged and had clumps of cells attached to them. Using the formula [(Number of dead larvae/Total number of larvae) × 100] the % killing of larvae was calculated.

### In vitro proliferation of PBMCs in presence of recombinant proteins and mitogens

PBMCs of EN, MF and CP subjects were cultured in 96-well round-bottomed microtiter plates (0.2 × 10^5^ cells/well in C-RPMI 1640). Cells used as positive controls were stimulated at 37 °C with Concanavaline A (ConA, 5 μg/ml). Experimental wells contained 10 μg/ml of Bm-TPP or BmAF-Myo or 20 μg/ml of Wol T1 IF-1 or *w*Bm-LigA. Wells containing medium with cells only, served as negative controls. Optimum concentration of recombinant proteins and mitogens were previously determined by exposing PBMCs of a normal healthy volunteer to various concentrations (1 to 20 μg/ml) of each recombinant protein. Cells were incubated for 72 h in case of proteins, while ConA was done for only 48 h. All cultures were done in triplicate, and cell proliferation was determined by MTT assay using the formula: stimulation index (SI) = absorbance of recombinant stimulated cells/absorbance of unstimulated cells. The stimulation indices indicate the cellular immune response of human population to the recombinant protein.

### Reactive oxygen species (ROS) detection

PBMCs (0.2 × 10^5^ cells/well) were cultured in triplicates for 48 h with recombinant proteins at the same concentration as for PBMC proliferation in CO_2_ incubator at 37 °C. After incubation, PBMCs were scrapped and washed twice with PBS. Finally, dichloro-dihydro-fluorescein diacetate DCFH-DA (10 μM) dye was added to the cells, which were then incubated at 37 °C for 30 min in dark. Cells were washed, suspended in PBS, and kept on ice for an immediate detection by flow cytometry. Data were acquired by FACS Conto II (Becton Dickinson, San Jose, CA) and analyzed using the FlowJo software [35].

### Statistical analysis

Data were summarized as means ± SD (standard deviation). Data were subjected to non-parametric analysis after ascertaining normality by Shapiro-Wilk’s test and homogeneity of variances between the groups by Levene’s test. Groups were compared by Kruskal-Wallis ANOVA and the significance of the differences in mean ranks between groups was assessed by Mann-Whitney U-test (adjusted *Z-*value and *P*-value). A two-tailed *P* < 0.05 was considered statistically significant. Analysis were performed on STATISTICA software (Windows version 7.1, Stat Soft, Inc., USA). Test statistics with exact *P*-values are presented in Additional file 2: Tables S2-S14.

## Results

### All four proteins were successfully overexpressed and purified by affinity chromatography

All four recombinant proteins were overexpressed and purified by Ni-NTA column. Bm-TPP, BmAF-Myo, Wol Tl IF-1 and *w*Bm-LigA were eluted in phosphate/Tris (wBm-LigA) buffer using 250 mM imidazole concentration. Single bands were obtained on 10% SDS PAGE confirming the presence of purified recombinant proteins in eluted fraction. Molecular masses of Bm-TPP, BmAF-Myo, Wol Tl IF-1 and *w*Bm-LigA were ~60kDa, ~73kDa, ~13kDa and ~75kDa, respectively (Additional file 3: Figure S1).

### Reactivity of *W. bancrofti* serum antibodies with *B. malayi* and *Wolbachia* recombinant proteins

**Fig. 1:**
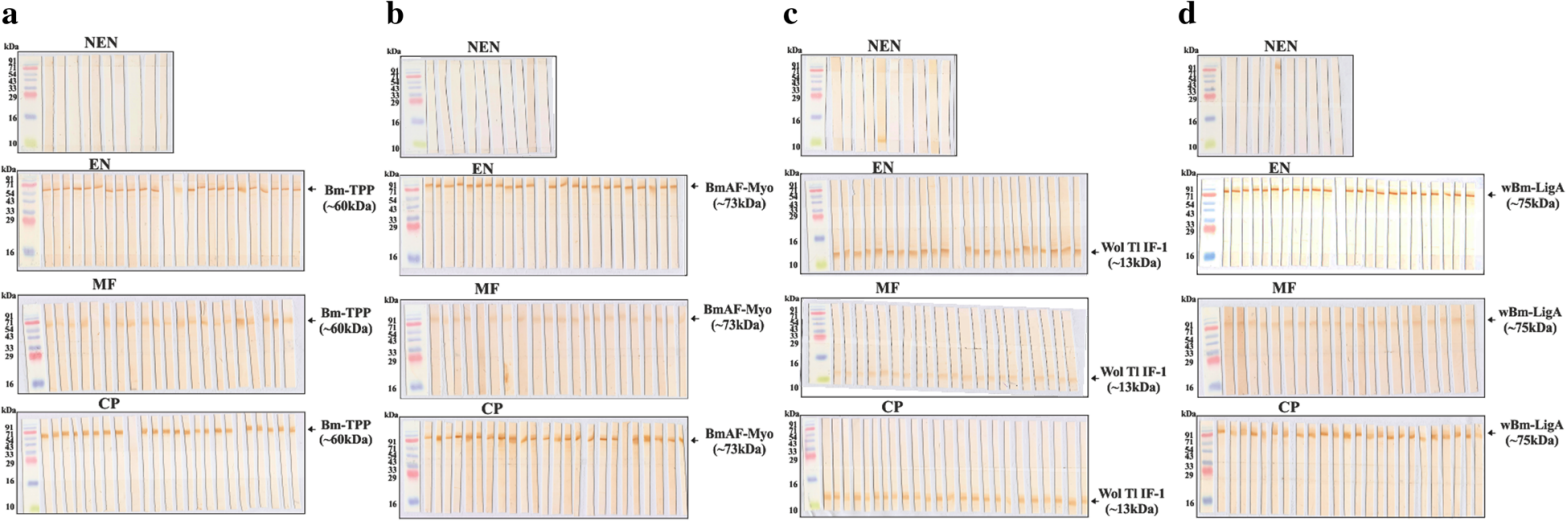
Immunoblotting of recombinant proteins with serum samples of various bancroftian categories. All serum samples of NEN (*n* = 10), EN (*n* = 24), MF (*n* = 21) and CP (*n* = 24) categories were checked for the presence of **a** anti-Bm-TPP antibodies; **b** antibodies against recombinant BmAF-Myo; **c** anti-Wol Tl IF-1 antibodies; and **d** antibodies against recombinant *w*Bm-LigA. Each strip represents immunoreactivity of individual serum sample with recombinant *B. malayi* and *Wolbachia* proteins. First strip in each group represents a standard molecular weight marker (PG-PMT2922, Puregene Genetix)

Seventy nine human serum samples including NEN (10), EN (24), MF (21) and CP (24) were tested individually for antibody reactivity with all the four recombinant proteins in Western blotting using anti-human IgG-HRP as secondary antibody. None of the ten NEN sera demonstrated any IgG antibody reactivity with Bm-TPP or BmAF-Myo, and only one reacted with Wol Tl IF-1 and *w*Bm-LigA. Twenty-three of the 24 EN sera reacted positively with all the four proteins. In the 21 MF samples, 20 were positive for anti-Bm-TPP and anti-BmAF-Myo antibodies, while all 21 samples showed reactivity with Wol Tl IF-1 and *w*Bm-LigA in blots. All of the 24 CP sera were positive for anti-Wol Tl IF-1 and anti-*w*Bm-LigA antibodies, but BmAF-Myo reacted with only 23 of these and Bm-TPP with only 22. Among the clinical groups, MF category consistently exhibited low IgG reactivity resulting into low band intensity. IgG antibody reactivity to Wol Tl IF-1 was also not as intense as with the other three proteins including *w*Bm-LigA. Thus, overall comparison of the antibody reactivity to both *B. malayi* and *Wolbachia* proteins revealed that human subjects staying in *W. bancrofti* endemic area are exposed to Bm-TPP, BmAF-Myo, Wol Tl IF-1 and *w*Bm-LigA proteins and generated substantial IgG antibodies to these antigens. The reactivity was specific; non-specific reaction was observed in only one NEN individual who showed reactivity with both the *Wolbachia* recombinant proteins (Fig. 1a-d; Table 1).

**Table 1:** Immunoblotting of recombinant proteins with clinical sera samples

Clinical category	Bm-TPP	BmAF-Myo	Wol Tl IF-1	*w*Bm-LigA
	No. of positive samples/Total no. of samples	No. of positive samples/Total no. of samples	No. of positive samples/Total no. of samples	No. of positive samples/Total no. of samples
NEN	0/10	0/10	1/10	1/10
EN	23/24	23/24	23/24	23/24
MF	20/21	20/21	21/21	21/21
CP	22/24	23/24	24/24	24/24

### Endemic normal population have high IgG antibody titer to the four proteins studied

IgG reactivity was assessed by measuring the antibody titer in pooled human sera of each category including NEN control. Using 2-fold serial dilution of pooled sera, 8-fold higher IgG antibody titers were detected against BmAF-Myo or Bm-TPP in EN (1/6,400) as compared to CP or MF (1/800). On the other hand, IgG levels were lower but similar against both the *Wolbachia* recombinant proteins (1/800) in all the three categories. Thus, it was apparent that EN population produced higher specific-IgG levels to *B. malayi* proteins than other clinical categories while all the clinical categories of patients contained IgG antibodies to *Wolbachia* proteins though the titer was comparatively low (Additional file 4: Figure S2).

**Fig. 2:**
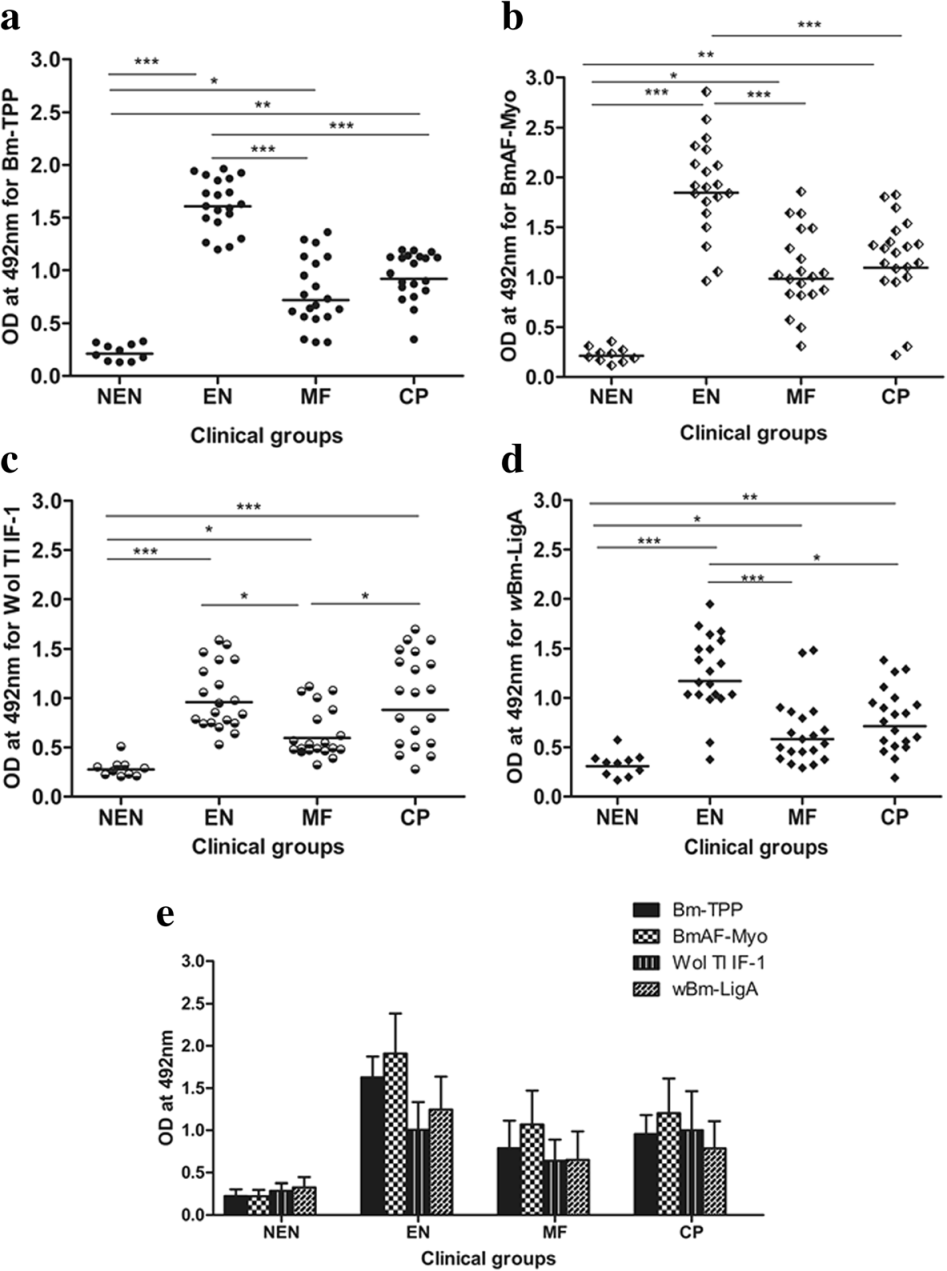
IgG antibody levels in bancroftian sera against recombinant *B. malayi* and *Wolbachia* proteins. IgG antibodies in each individual of different categories were measured by ELISA for **a** Bm-TPP; **b** BmAF-Myo; **c** Wol Tl IF-1; and **d** *w*Bm-LigA proteins and presented in scatter plots where each dot represents absorbance of individual sera and horizontal lines represent the mean. **e** Bar graphs showing protein levels (mean + standard deviation) in each group. Groups were compared using Kruskal-Wallis ANOVA and the significance of the differences in mean ranks between groups was assessed by Mann-Whitney U test. Data are from one of the three similar experiments using same serum sample. ^*^ *P* < 0.05; ^**^ *P* < 0.01; ^***^ *P* < 0.001

Further, all serum samples were individually tested in indirect ELISA at 1/800 dilution after coating the plate with four recombinant proteins to observe the differential specific IgG response. IgG antibody level for Bm-TPP was significantly higher in EN individuals than in the CP and MF group. Likewise, with the BmAF-Myo and *w*Bm-LigA, the EN category again showed high OD values, while no significant difference was observed in MF and CP categories. In general, antibody responses were lowest in MF group, although even in this group the values were significantly higher than NEN group. Wol Tl IF-1 had the same level of IgG reactivity in EN and CP group, a level significantly higher than MF group. The combined graph clearly shows that in EN category, *B. malayi* recombinant proteins (Bm-TPP and BmAF-Myo) exhibited higher serum reactivity than *Wolbachia* proteins (Wol Tl IF-1 and *w*Bm-LigA). IgG level observed in this population was highest for BmAF-Myo (Fig. 2a-e).

### Isotype antibody response

**Fig. 3:**
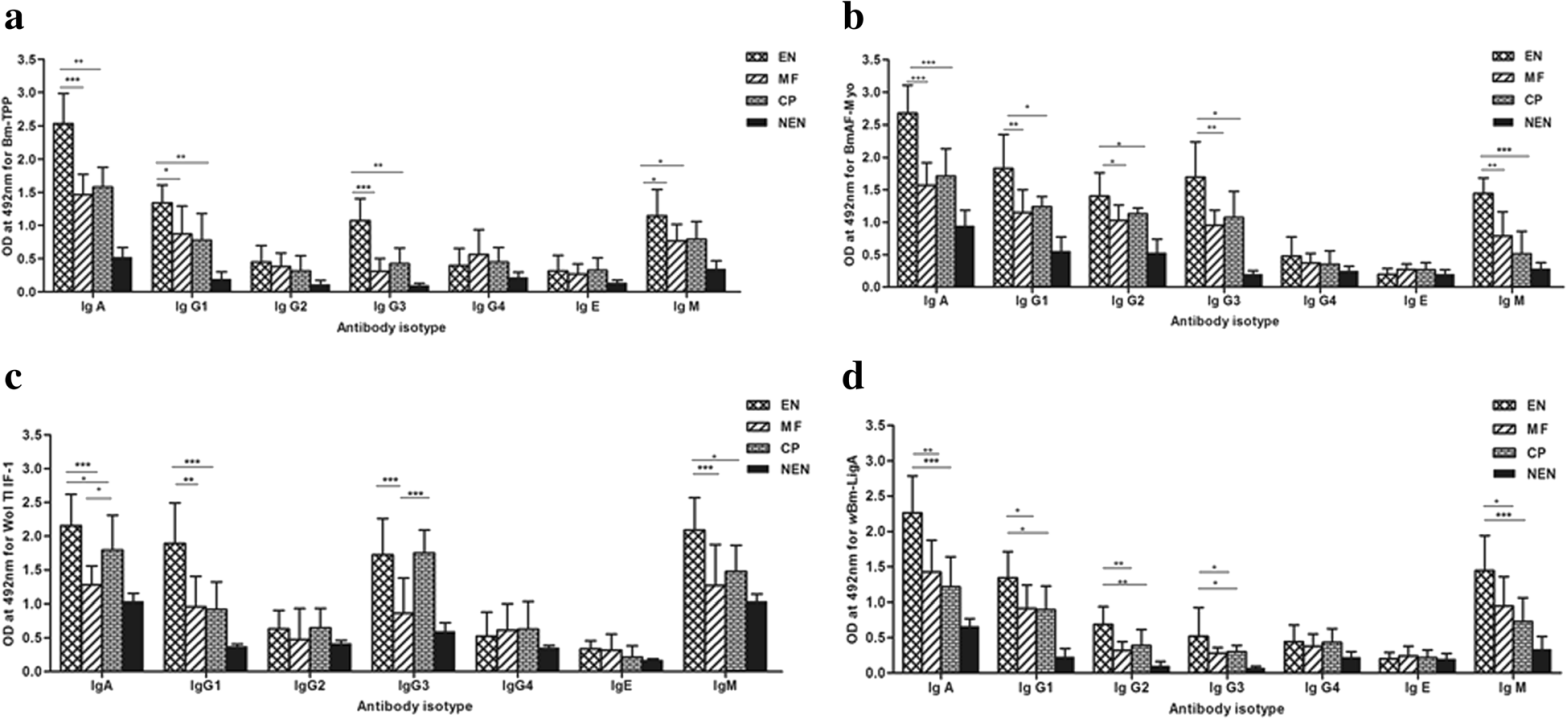
Antibody isotypes in sera of *Wuchereria bancrofti*-exposed humans to recombinant proteins. IgA, IgG1, IgG2, IgG3, IgG4, IgE and IgM isotype antibodies in various groups (EN, MF, CP and NEN) for **a** Bm-TPP; **b** BmAF-Myo; **c** Wol Tl IF-1; and **d** *w*Bm-LigA proteins, measured by ELISA after coating the plate with 0.1 μg/ml of each protein antigen. Data (OD values corresponding to antibody isotype reactivity of serum of all individuals of a group for each protein) are summarized as mean + standard deviation (NEN, *n* = 10; EN, MF and CP, *n* = 20). For each protein, comparison between groups was performed by Kruskal-Wallis ANOVA and the significance of the differences in mean ranks between groups was assessed by Mann-Whitney U test. Data are from one of the three similar experiments using same serum sample. ^*^ *P* < 0.05; ^**^ *P* < 0.01; ^***^ *P* < 0.001

Humoral immune response was further investigated by analyzing the antibody isotypes in sera of the same groups (EN, MF, CP and NEN control) reacting with *B. malayi* and *Wolbachia* recombinant proteins. IgA, IgG1, IgG3 and IgM isotypes were predominantly and significantly higher in EN subjects than other categories for Bm-TPP while no significant differences were observed for IgG4, IgG2 and IgE. BmAF-Myo more strongly reacted with the antibodies present in the EN sera showing high IgA, IgG1, IgG2, IgG3 and IgM, but no significant differences were observed in IgG4 and IgE levels among the four categories. Regarding Wol Tl IF-1-specific antibody isotype levels, reactivity was higher for IgA and IgG3 in EN and CP category as compared to MF and NEN individuals. IgG1 and IgM were high in EN category while no group showed significant changes in IgG4 and IgE levels. *w*Bm-LigA showed similar isotype reactivity as in BmAF-Myo, although the antibody levels were lower. Overall isotype investigation revealed that IgA and IgM isotypes were higher in EN category irrespective of recombinant protein used in ELISA (Fig. 3a-d).

### Antibodies to recombinant proteins in bancroftian sera contribute to L3 killing *via* ADCC

**Fig. 4:**
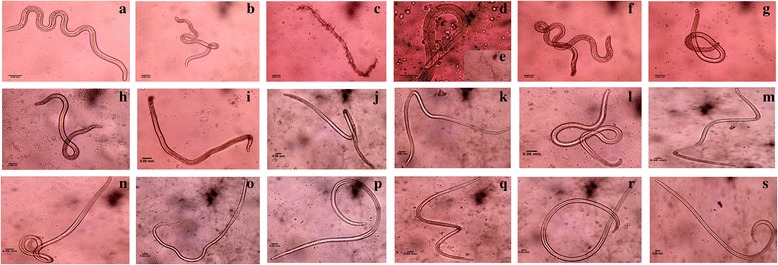
L3 larvae of *B. malayi* recovered from cultures after antibody dependent cellular cytotoxicity assay. L3 were incubated with PBMCs and **a** without EN sera (negative control); **b** with NEN pooled sera; **c-e** with pooled EN sera; **f** with pooled EN sera after depleting anti Bm-TPP antibodies; **g** with anti-BmAF-Myo antibodies depleted EN pooled sera; **h** with anti-Wol Tl IF-1 antibodies depleted EN pooled sera; **i** with anti-*w*Bm-LigA antibodies depleted EN sera; **j** with pooled MF sera; **k** with pooled MF sera after depleting anti Bm-TPP antibodies; **l** with anti-BmAF-Myo antibodies depleted MF pooled sera; **m** with anti-Wol Tl IF-1 antibodies depleted MF pooled sera; **n** with anti-*w*Bm-LigA antibodies depleted MF sera; **o** with pooled CP sera; **p** with pooled CP sera after depleting anti-Bm-TPP antibodies; **q** with anti-BmAF-Myo antibodies depleted CP pooled sera; **r** with anti-Wol Tl IF-1 antibodies depleted CP pooled sera; and **s** with anti-*w*Bm-LigA antibodies depleted CP sera. Photographs were captured on a phase contrast microscope (Nikon, Japan). *Scale-bars*: 0.05 mm in all panels

Individuals residing in endemic area of bancroftian filariasis continuously get exposed to L3, triggering formation of antibodies. These antibodies participate in larvae killing *via* ADCC mechanism, and have a role in providing immunity to EN individuals. In vitro ADCC assay was carried out to observe cellular adherence, cytotoxicity and larval killing in the presence of patients’ sera containing antibodies to four recombinant proteins. These findings revealed that antibodies present in pooled EN sera participated more actively in cellular adherence and caused parasite death within 48 h, as compared to antibodies present in MF and CP pooled sera. The differential role of protein-specific antibodies was investigated by their depletion and incubating the L3 with antibody-depleted EN/MF/CP pooled sera. Cellular adherence and subsequent cytotoxic killing of larvae was reduced profoundly after depletion of anti-Bm-TPP and anti-*w*Bm-LigA antibodies especially in EN individuals. The other proteins also exhibited reduced ADCC, but to a lesser degree which was also not significant (Fig. 4a-s; Table 2).

**Table 2:** Results of ADCC assay against *B. malayi* L3 using human sera

No.	Groups	% Killing (mean ± SD)
1	Control (L3 + PBMCs)	0
2	NEN (L3 + PBMCs + Pooled NEN sera)	9.7 ± 4.0
3	EN (L3 + PBMCs + Pooled EN sera)	84.1 ± 3.6^a^
4	L3 + PBMCs + anti Bm-TPP antibodies depleted Pooled EN sera	30.2 ± 3.0^b^
5	L3 + PBMCs + anti BmAF-Myo antibodies depleted Pooled EN sera	51.1 ± 1.9
6	L3 + PBMCs + anti Wol Tl IF-1 antibodies depleted Pooled EN sera	47.6 ± 4.1
7	L3 + PBMCs + anti *w*Bm-LigA antibodies depleted Pooled EN sera	29.4 ± 4.2^b^
8	MF (L3 + PBMCs + Pooled MF sera)	31.5 ± 1.6
9	L3 + PBMCs + anti Bm-TPP antibodies depleted Pooled MF sera	21.8 ± 1.6
10	L3 + PBMCs + anti BmAF-Myo antibodies depleted Pooled MF sera	22.9 ± 4.9
11	L3 + PBMCs + anti Wol Tl IF-1 antibodies depleted Pooled MF sera	23.9 ± 3.4
12	L3 + PBMCs + anti *w*Bm-LigA antibodies depleted Pooled MF sera	22.7 ± 3.5
13	CP (L3 + PBMCs + Pooled CP sera)	35.0 ± 3.6
14	L3 + PBMCs + anti Bm-TPP antibodies depleted Pooled CP sera	23.4 ± 1.4
15	L3 + PBMCs + anti BmAF-Myo antibodies depleted Pooled CP sera	27.8 ± 2.9
16	L3 + PBMCs + anti Wol Tl IF-1 antibodies depleted Pooled CP sera	22.1 ± 2.6^b^
17	L3 + PBMCs + anti *w*Bm-LigA antibodies depleted Pooled CP sera	24.9 ± 1.8

Data are summarized as Mean ± SD, *n* = 3. The comparison were done between pooled EN/MF/CP sera and pooled EN/MF/CP sera after depletion of anti Bm-TPP/anti BmAF-Myo/Wol Tl IF-1/anti wBm-LigA antibodies

^a^ *P* < 0.05; significant differences between pooled EN sera with pooled MF sera and pooled CP sera

^b^ *P* < 0.05; significant differences of % killing in each group after depletions of protein-specific antibodies with respect to undepleted pooled sera

### *Brugia malayi* and *Wolbachia* recombinant proteins promote PBMC proliferation in vitro

**Fig. 5:**
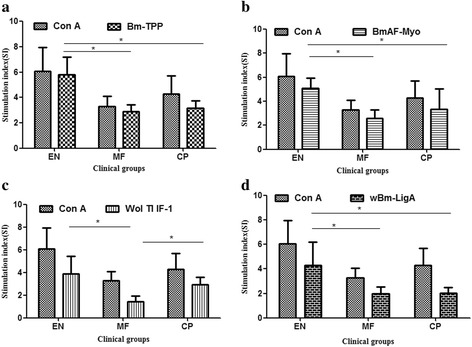
PBMC proliferation of human subjects. **a** PBMCs of EN, MF and CP stimulated with CoA (5 μg/ml) or Bm-TPP (10 μg/ml); **b** PBMCs stimulated with CoA (5 μg/ml) or BmAF-Myo (10μg/ml); **c** CoA (5 μg/ml) or Wol Tl IF-1 (20 μg/ml) stimulated PBMCs; **d** EN, MF and CP clinical samples’ PBMC stimulated with CoA (5 μg/ml) or *w*Bm-LigA (10 μg/ml). Proliferation was assessed as stimulation index (SI) by MTT assay. Data are from one of the three similar experiments and summarized as mean + standard deviation (*n* = 5). Groups were compared using Kruskal-Wallis ANOVA and the significance of the differences in mean ranks between groups was assessed by Mann-Whitney U test. ^*^ *P* < 0.05; ^**^ *P* < 0.01; ^***^ *P* < 0.001

PBMCs upon stimulation with an immunogen or mitogen undergo clonal proliferation of B-cells, T-cells and initiation of humoral and cellular immune response by monocytes connecting both arms of immunity. This cell-proliferation is considered as an indirect marker for predicting the immunogenic response of host to a particular antigen. The EN population responded more strongly to all the recombinant proteins than did MF and CP individuals, except Wol Tl IF-1 where stimulation was almost same in EN and CP categories. *Brugia malayi* proteins caused higher cell and similar proliferation (BmAF-Myo SI 5.99; Bm-TPP SI 5.79), which was statistically significant as compared to MF or CP groups. *w*Bm-LigA demonstrated similar effects with mean SI 4.31 ± 1.90 for EN group that was significantly higher than in the MF or CP categories. Wol Tl IF-1 also led to cell proliferation in PBMCs of EN subjects (mean SI 3.87) which was significantly higher than in the MF population but not in the CP group. Strong proliferation of cells isolated from EN subjects in response to Bm-TPP, BmAF-Myo and *w*Bm-LigA suggests the possible involvement of these proteins in protection mechanism that keeps EN population free from filarial infection. However, similar SI index of Wol Tl IF-1 in EN and CP categories may suggest involvement of this factor in protection and/or pathogenesis (Fig. 5a-d).

### Higher stimulation of ROS generation in EN subjects as compared to MF and CP category

**Fig. 6:**
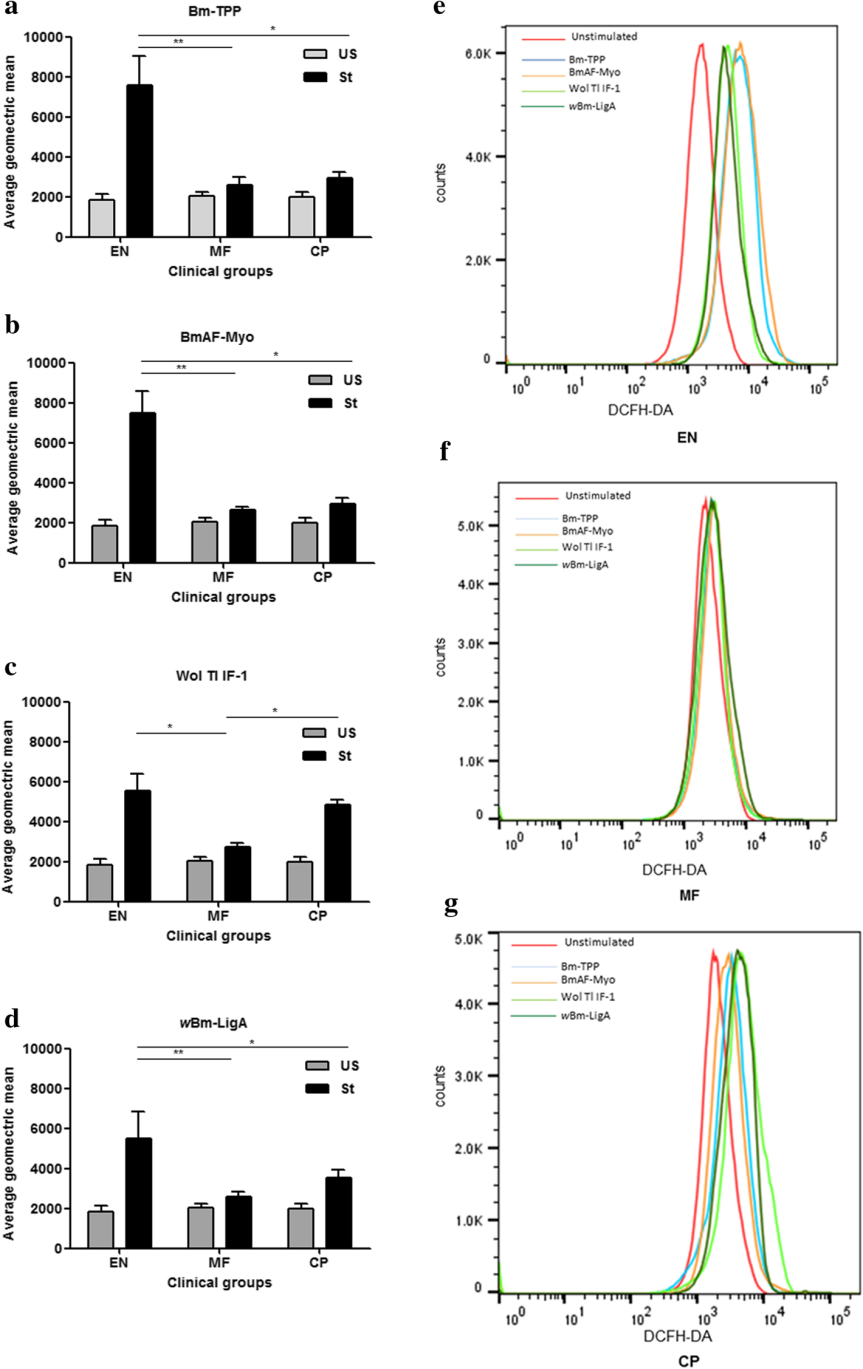
Oxidative burst in human PBMCs of bancroftian categories when stimulated with recombinant proteins. Reactive oxygen species (ROS) generation in PBMCs was measured by flow cytometry using DCF-DA after stimulating with recombinant proteins **a** Bm-TPP; **b** BmAF-Myo; **c** Wol Tl IF-1; and **d** *w*Bm-LigA. Unstimulated PBMCs (US) and stimulated PBMCs (St) data are summarized as mean + standard deviation (*n* = 5). Presentation of shifting of florescence peak of different proteins in (**e**) EN group; **f** MF group; and **g** CP group. Stimulated groups were compared using Kruskal-Wallis ANOVA and the significance of the differences in mean ranks between groups was assessed by Mann-Whitney U test. ^*^ *P* < 0.05; ^**^ *P* < 0.01; ^***^ *P* < 0.001

Our earlier findings on immunization of rodents with Bm-TPP and Wol Tl IF-1 have shown production of ROS by macrophages rich peritoneal cells [25, 28]. Here we analyzed oxidative burst in PBMCs of all clinical categories after stimulation with the recombinant proteins. Results demonstrate clear-cut shifting of average fluorescence intensity in EN group unlike MF or CP group and this was more prominent on stimulation with *B. malayi* recombinant proteins. Wol Tl IF-1, on the other hand, stimulated cells from both EN and CP categories to almost similar extent. Mean florescence of stimulated PBMCs for ROS for all three proteins was also significantly higher in EN individuals as compare to other categories. In Wol Tl IF-1-stimulated cells, nearly the same average geometric mean fluorescence was observed for EN and CP categories (Fig. 6a-g).

## Discussion

Bm-TPP and BmAF-Myo earlier showed good immunoprophylactic efficacy in experimental animal models in our laboratory [24–27]. We also cloned and characterized two *Wolbachia* proteins, Wol Tl IF-1 and *w*Bm-LigA [28–30]. In the current investigation, we undertook immune characterization of all the four recombinant proteins to assess both humoral and cellular immune response of human subjects staying in *W. bancrofti*-endemic areas. The IgG antibody response was evaluated both qualitatively and quantitatively in all categories of sera to understand humoral immune response of human host to above recombinant antigens. Bm-TPP and BmAF-Myo revealed specific reaction with bancroftian antibodies as all serum samples of filaria-free zone (NEN category) failed to show any reaction indicating that these proteins are well accessed by the human host. As EN population get continuously exposed to L3 and immune to infection, strong immunological responses of EN individuals to these proteins suggested that the proteins are likely to be derived primarily from infective larval stages. Nevertheless, adult and microfilariae do share these proteins as observed earlier [23, 24]. Reactivity of bancroftian IgG with recombinant Wol Tl IF-1 and *w*Bm-LigA was also filaria-specific; however, one of the ten sera in NEN category showed some reactivity in both blot and ELISA. The possible reason could be a cross-reaction with some bacteria or some other organism carrying an epitopic region similar to these wolbachial proteins. Death and disintegration of filarial worms has been suggested to expose host to proteins of *Wolbachia* that may anticipate higher reactivity of CP individuals to *Wolbachia* proteins; however, almost similar band intensity or ELISA antibody titer observed in EN sera suggests the presence of anti-wolbachial antibodies in EN population as well. As these subjects are continuously exposed to infective larval invasion and larvae do not normally develop further and die thus may release *Wolbachia* intermittently. Strong reactivity of all the four recombinant proteins with EN sera points towards the immunoprotective nature of these proteins. IgG titers to *B. malayi* antigens were higher than those of *Wolbachia* antigens also suggests that *B. malayi* antigens could be a better candidate for vaccine as compare to *Wolbachia* antigens. Earlier, few recombinant *B. malayi/W. bancrofti* proteins have been proposed as vaccine candidates based on their strong IgG reactivity with EN individuals [33, 36, 37]. Apart from IgG antibody, protein specific serum IgA and IgM were also in higher concentration in EN group while all MF individuals contained low antibody reactivity and CP had moderate. A protective role for IgA has been reported for several helminthic infections such as *Schistosoma mansoni*, *Taenia taeniformis*, *Trichinella spiralis*, including cattle filarial parasite *Setaria digitata* [38–41]. Protective role of IgM has also been postulated in *Strongyloides stercoralis*, *Brugia phangi* and *W. bancrofti* infections [37, 42, 43]*.*

Previous studies in our laboratory in rodent models illustrated that Bm-TPP and Wol Tl IF-1 generated a mixed Th1/Th2 and Th2 biased immune responses respectively [26, 28]. However, in the current study human EN population showed a Th2 biased immune response for Bm-TPP and Wol Tl IF-1 with predominant increase in IgG1 and IgG3 isotype. IgG1, IgG2 and IgG3 isotype levels were found to be higher in EN population against BmAF-Myo and *w*Bm-LigA recombinant proteins. In several studies involving various recombinant filarial proteins, elevated levels of IgG1, IgG2 and IgG3 isotypes have been reported in putatively immune individuals [14, 33, 36, 44, 45] suggesting their role in filarial larval killing. Nevertheless, polarization of T-helper cell response in EN category towards type 1 or type 2 cannot be specifically be mentioned since it depends upon the type of protein antigen [36, 45]. IgG4 is a marker of active filarial infection [46, 47] and not much variation in the level of this antibody subclass in any clinical category of human subjects was noticed for individual proteins. In CP individuals, IgG3 antibodies against Wol Tl IF-1 were found to be significantly higher than in MF group (though lower than EN) indicating exposure of CP and EN both population to Wol Tl IF-1 protein. The role of this factor in the development of pathology/protection in filarial infection needs to be resolved before this protein is considered further for vaccination experimentations. IgG isotyping data also suggest prospective immunogenicity of Bm-TPP and BmAF-Myo; however, more studies are required to explore the functions of *Wolbachia* proteins.

IgG1 and IgG3 are cytophilic antibodies that bind to FcγRI and RII receptors expressed on macrophages, neutrophils, eosinophils and mediate parasite killing *via* ADCC mechanism [48]. Amongst all three categories, EN serum pool brought about highest cytotoxicity and parasite mortality with respect to MF and CP serum pool. In vitro killing of *B. malayi* L3 in presence of EN sera is likely to be mediated by these two IgG subclasses that were abundant in EN patients and promoted cellular adherence and cytotoxicity. This ability was lost once the EN sera were adsorbed with proteins in which Bm-TPP and *w*Bm-LigA, substantiating their role in larval killing and correlated with their profound immunoprophylactic efficacy [26]. Enhanced proliferative response of Bm-TPP, BmAF-Myo and *w*Bm-LigA suggested the role of these proteins in inducing cellular immune response; however, Wol Tl IF-1 needs careful investigation as vaccine candidate owing to its strong reactivity with CP antibodies and inducing cell proliferation in CP patients. All four recombinant antigens show T-cell hyporesponsiveness in MF group as the SI values were found to be consistently low. The proteins demonstrated both antigen-specific and non-specific hyporesponsiveness similar to other immunogens like Con A. Few other filarial antigens have also been reported to respond in the same manner with EN patients [34, 44]. *Brugia malayi* proteins stimulated PBMCs generate ROS in EN population though proteins from *Wolbachia* did not participate to that extent. However, Wol Tl IF-1 induces ROS in both EN and CP categories. These findings thus further substantiate a protective role of all the four recombinant proteins; nevertheless, Wol Tl IF-1 may also induce immunopathological responses that need to be explored further. We earlier mentioned that in vitro killing of both L3 and microfilariae is mediated *via* IFN-γ activated macrophages through ROS generation on immunization of animals with Bm-TPP and Wol Tl IF-1 these previous findings support current observations [25, 28].

The correlation between responses of serum category with the type of recombinant protein was also revealed in the current investigation. The ADCC-mediated killing of L3 clearly shows that EN antibodies play an important role in killing of larval *B. malayi* since the phenomenon was highest in this serum category. Amongst all, Bm-TPP and *w*Bm-LigA revealed best results with EN sera as far as ADCC is concerned. These results could be correlated with our previous animal studies. IgG, isotypes, cellular proliferation and ROS generation also indicate possible role of these two proteins in the host at early infection. Individuals of MF group are hypo-responsive and therefore their responses were minimal with any recombinant protein including these two. Wol Tl IF-1 had more or less similar IgG and cellular response in EN and CP categories, and might therefore have some role in host pathology. *Wolbachia* has been suggested to play some role in filarial pathology. The removal of anti-Bm-TPP and anti-*w*Bm-LigA from EN sera by immunoadsorption led to significant decrease in cell adherence and larval toxicity. On the other hand, depletion of serum antibodies to BmAF-Myo reduced the ADCC-mediated killing by half which was not significant but other results of this study with BmAF-Myo also point towards the similar role as that of Bm-TPP and *w*Bm-LigA. These are preliminary findings and show that Bm-TPP, *w*Bm-LigA and BmAF-Myo recombinant proteins possibly become exposed to host during very early infection stages while Wol Tl IF-1 also play some role in host pathology.

Altogether, our study shows that a putatively immune population in an endemic area carries antibodies not only against *B. malayi* recombinant proteins but also against its endosymbiont antigens. It is reported that adult worm death results into *Wolbachia* release and most of the *Wolbachia* proteins participate in inflammatory responses [49]. Wol Tl IF-1 is released as ES products by live adult *B. malayi* and a recent report indicates that the vertebrate host becomes exposed to *Wolbachia* or their products that are released through the lumen of the secretory-excretory canals of *B. malayi* [50]. Reports do exist for the presence of *Wolbachia* at all life stages [51, 52] and therefore *Wolbachia* release in EN subjects as a result of repeated larval exposure and their subsequent killing is justified.

## Conclusions

Overall, this study explores immune characterization of Bm-TPP, BmAF-Myo, Wol Tl IF-1 and *w*Bm-LigA recombinant proteins in filarial endemic population. Our findings, along with previous reports, support that these *B. malayi* recombinant proteins might be used as a potential vaccine candidate against bancroftian filariasis. The presence of anti-*Wolbachia* antibodies in the EN population indicates that *Wolbachia* proteins may also play important role during the early stages of infection; however to validate this observation more research inputs are needed. These findings are noteworthy in view of non-availability of a drug or vaccine to target adult filarial parasites.

## Additional files

Additional file 1: Table S1. Detailed information of the individuals of bancroftian filariasis endemic area participated in the current study. (PDF 54 kb)
Additional file 2: Table S2. Statistical analysis of IgG among different groups (NEN, EN, MF and CP) for each protein. **Table S3.** Statistical analysis of IgA among different groups (NEN, EN, MF and CP) for each protein. **Table S4.** Statistical analysis of IgG1 among different groups (NEN, EN, MF and CP) for each protein. **Table S5.** Statistical analysis of IgG2 among different groups (NEN, EN, MF and CP) for each protein. **Table S6.** Statistical analysis of IgG3 among different groups (NEN, EN, MF and CP) for each protein. **Table S7.** Statistical analysis of IgG4 among different groups (NEN, EN, MF and CP) for each protein. **Table S8.** Statistical analysis of IgE among different groups (NEN, EN, MF and CP) for each protein. **Table S9.** Statistical analysis of IgM among different groups (NEN, EN, MF and CP) for each protein. **Table S10.** Statistical analysis of PBMC proliferation between groups (EN, MF and CP) for each protein. **Table S11.** Statistical analysis of ROS generation in terms of mean fluorescence for each protein, comparison of stimulated PBMC between groups (EN, MF and CP) were done. **Table S12.** Statistical analysis, comparison of % killing (Mean ± SD, *n* = 3) between three groups at baseline (no depletion of protein specific antibodies) using Kruskal-Wallis ANOVA. **Table S13.** Multiple comparisons of mean ranks of % killing between three groups at baseline. **Table S14.** Statistical analysis; For each group, comparisons of % killing between different proteins after depletion of protein specific antibodies at baseline. (PDF 118 kb)
Additional file 3: Figure S1. Overexpression and purification of *B. malayi* and *Wolbachia* recombinant proteins. **a** Coomassie blue stained SDS-polyacrylamide gel. Lane M: standard protein molecular weight marker (Thermoscientific 815-968-0747); Lane 1: soluble *E. coli* lysates; Lane 2: flow through; Lane 3: wash through prior to elution; Lane 4: purified recombinant Bm-TPP. **b** Lane M: standard protein molecular weight marker; Lane 1: soluble *E. coli* lysates after sonication; Lane 2: flow through; Lane 3: wash through; Lane 4: purified recombinant BmAF-Myo. **c** Lane M: standard protein molecular weight marker; Lane 1: *E. coli* cell lysates supernatant; Lane 2: flow through; Lane 3: wash through prior to elution; Lane 4: purified eluted recombinant Wol Tl IF-1. **d** Lane M: standard protein molecular weight marker; Lane 1: soluble cell lysates; Lane 2: flow through; Lane 3:wash through; Lane 4: purifiied eluted *w*Bm-LigA. (TIF 10119 kb)
Additional file 4: Figure S2. IgG antibody titer of human bancroftian pateints of different clinical groups and non-filarial individuals. IgG antibody titer values of each category for each protein were evaluated *via* ELISA by 2-fold serial dilution of pooled human sera that was used as primary antibody. Graph between mean OD values at 492 nm of triplicate wells along with standard errors are plotted on the y-axis against dilution factor along the x-axis for **a** Bm-TPP; **b** BmAF-Myo; **c** Wol Tl IF-1; and **d** *w*Bm-LigA. (TIF 9547 kb)

## Abbreviations

ADCC: Antibody dependent cell mediated cytotoxicity
BmAF-Myo: *Brugia malayi* adult female heavy chain myosin
Bm-TPP: *Brugia malayi* trehalose-6-phosphate phosphatase
CDRI: Central Drug Research Institute
CFA: Circulating filarial antigen
CO_2_: Carbon dioxide
ConA: Concanavaline A
CP: Chronic patients
CPCSEA: Committee for the Purpose of Control and Supervision of Animals
C-RPMI1640: Complete- Roswell Park Memorial Institute medium 1640
CSIR: Council of Scientific & Industrial Research
DAB: 3,3′-diaminobenzidine tetra hydrochloride
DCFH-DA: Dichloro-dihydro-fluorescein diacetate
EC: Ethical clearance
ELISA: Enzyme-linked immunosorbent assay
EN: Endemic normal
EU: Endotoxin units
FACS: Fluorescence-activated cell sorting
FBS: Fetal bovine serum
H_2_O_2_: Hydrogen peroxide
H_2_SO_4_: Sulfuric acid
HRP: Horseradish peroxidase
IAEC: Institutional Animal Ethics Committee
ICT: Immunochromatographic card test
IFN-γ: Interferon- γ
Ig: Immunoglobulin
IPTG: Isopropyl β-D-1-thiogalactopyranoside
IRB: Internal Research and Review Board
kDa: Kilodaltons
LAL: Limulus amebocyte lysate
LF: Lymphatic filariasis
LPS: Lipopolysaccharide
MF: Microfilaraemic
MKU: Madurai Kamraj University
MTT: 3-(4, 5-dimethythiazol- 2-yl)-2,5-diphenyl tetrazolium bromide
NCBI: National Center for Biotechnology Information
NEN: Non-endemic normal
Ni-NTA: Nickel nitrilotriacetic acid
OD: Optical density
OPD: Orthophenylenediamine
PBMC: Human peripheral blood mononuclear cells
PBS: Phosphate buffer saline
PBST: Phosphate buffer saline tween-20
PCR: Polymerase chain reaction
PHC: Primary health center
PMSF: Phenylmethylsulfonyl fluoride
PVDF: Polyvinylidene difluoride
ROS: Reactive oxygen species
RPMI 1640: Roswell Park Memorial Institute medium 1640
SD: Standard deviation
SDS-PAGE: Sodium dodecyl sulfate-polyacrylamide gel electrophoresis
SI: Stimulation index
Th: T helper
Tris: Tris(hydroxymethyl)aminomethane
*w*Bm-LigA: *Wolbachia* NAD^+^-dependent DNA ligase
Wol Tl IF-1: *Wolbachia* translation initiation factor-1
