# Supplementary material for: Humans from Wuchereria bancrofti endemic area elicit substantial immune response to proteins of the filarial parasite Brugia malayi and its endosymbiont Wolbachia
Source: Parasit Vectors. 2017 Jan 24;10:40. doi: 10.1186/s13071-016-1963-x (PMC5259955; doi:10.1186/s13071-016-1963-x)
Supplement: Additional file 1: Table S1. — Detailed information of the individuals of bancroftian filariasis endemic area participated in the current study. (PDF 54 kb) [file 13071_2016_1963_MOESM1_ESM.pdf]

Additional file 1

Table S1. Detailed information of the individuals of bancroftian filariasis endemic area participated in the current study.

S.No.	Clinical category	Age	Gender	Mf Status	Rounds of MDA
**1.**	EN	35	M	-ve	
**2.**	EN	38	M	-ve	
**3.**	EN	40	F	-ve	
**4.**	EN	53	F	-ve	
**5.**	EN	42	M	-ve	
**6.**	EN	33	F	-ve	
**7.**	EN	47	M	-ve	
**8.**	EN	52	M	-ve	
**9.**	EN	28	F	-ve	
**10.**	EN	31	M	-ve	
**11.**	EN	38	M	-ve	
**12.**	EN	32	M	-ve	
**13.**	EN	29	F	-ve	
**14.**	EN	30	M	-ve	
**15.**	EN	37	F	-ve	
**16.**	EN	33	M	-ve	
**17.**	EN	41	F	-ve	
**18.**	EN	40	M	-ve	
**19.**	EN	26	F	-ve	
**20.**	EN	29	M	-ve	
**21.**	EN	37	M	-ve	5 rounds of MDA completed
**22.**	EN	36	F	-ve	
**23.**	EN	41	M	-ve	
**24.**	EN	44	M	-ve	
**25.**	MF	28	M	+ve	
**26.**	MF	35	F	+ve	
**27.**	MF	32	F	+ve	
**28.**	MF	27	M	+ve	
**29.**	MF	37	F	+ve	
**30.**	MF	38	M	+ve	
**31.**	MF	21	M	+ve	
**32.**	MF	48	M	+ve	
**33.**	MF	44	M	+ve	
**34.**	MF	50	M	+ve	
**35.**	MF	52	M	+ve	
**36.**	MF	34	F	+ve	
**37.**	MF	33	M	+ve	
**38.**	MF	57	M	+ve	
**39.**	MF	48	F	+ve	
**40.**	MF	29	F	+ve	
**41.**	MF	38	M	+ve	
**42.**	MF	33	M	+ve	
**43.**	MF	37	F	+ve	
**44.**	MF	45	M	+ve	
**45.**	MF	59	F	+ve	

**46.**	CP	28	M	-ve	
**47.**	CP	33	F	-ve	
**48.**	CP	37	M	-ve	
**49.**	CP	39	M	+ve	
**50.**	CP	30	F	-ve	
**51.**	CP	41	F	-ve	
**52.**	CP	48	F	-ve	
**53.**	CP	53	M	-ve	
**54.**	CP	55	M	-ve	
**55.**	CP	38	M	+ve	
**56.**	CP	47	M	-ve	
**57.**	CP	44	F	-ve	
**58.**	CP	29	M	-ve	
**59.**	CP	43	M	-ve	
**60.**	CP	45	M	-ve	
**61.**	CP	55	F	-ve	
**62.**	CP	58	M	-ve	
**63.**	CP	34	M	-ve	
**64.**	CP	31	M	-ve	
**65.**	CP	37	F	+ve	
**66.**	CP	64	M	-ve	
**67.**	CP	66	M	-ve	
**68.**	CP	54	M	-ve	
**69.**	CP	48	F	-ve	

Note:

EN = Endemic Normal; MF = Microfilaraemic; CP = Chronic patients; MDA = Mass Drug Administration; Mf = Microfilariae.
