# Supplementary material for: Humans from Wuchereria bancrofti endemic area elicit substantial immune response to proteins of the filarial parasite Brugia malayi and its endosymbiont Wolbachia
Source: Parasit Vectors. 2017 Jan 24;10:40. doi: 10.1186/s13071-016-1963-x (PMC5259955; doi:10.1186/s13071-016-1963-x)
Supplement: Additional file 2: Table S2. — Statistical analysis of IgG among different groups (NEN, EN, MF and CP) for each protein. Table S3. Statistical analysis of IgA among different groups (NEN, EN, MF and CP) for each protein. Table S4. Statistical analysis of IgG1 among different groups (NEN, EN, MF and CP) for each protein. Table S5. Statistical analysis of IgG2 among different groups (NEN, EN, MF and CP) for each protein. Table S6. Statistical analysis of IgG3 among different groups (NEN, EN, MF and CP) for each protein. Table S7. Statistical analysis of IgG4 among different groups (NEN, EN, MF and CP) for each protein. Table S8. Statistical analysis of IgE among different groups (NEN, EN, MF and CP) for each protein. Table S9. Statistical analysis of IgM among different groups (NEN, EN, MF and CP) for each protein. Table S10. Statistical analysis of PBMC proliferation between groups (EN, MF and CP) for each protein. Table S11. Statistical analysis of ROS generation in terms of mean fluorescence for each protein, comparison of stimulated PBMC between groups (EN, MF and CP) were done. Table S12. Statistical analysis, comparison of % killing (Mean ± SD, n = 3) between three groups at baseline (no depletion of protein specific antibodies) using Kruskal-Wallis ANOVA. Table S13. Multiple comparisons of mean ranks of % killing between three groups at baseline. Table S14. Statistical analysis; For each group, comparisons of % killing between different proteins after depletion of protein specific antibodies at baseline. (PDF 118 kb) [file 13071_2016_1963_MOESM2_ESM.pdf]

Additional File 2

<u>Statistical Analysis of Figures shown in graphs (Figure 2, 3, 5, 6).</u>

Analysis of Fig. 2 IgG antibody levels in bancroftian sera against recombinant *B. malayi* and *Wolbachia* proteins.

Table S2. For each protein, comparison between groups (NEN, EN, MF and CP) were done by Kruskal-Wallis ANOVA and significance of difference in mean ranks between groups was done by Mann-Whitney U test (Z value and p value).

**Comparisons**	**Bm-TPP**		**BmAF-Myo**		**Wol TI IF-1**		***w*****Bm-LigA**	
	**Z value**	**p value**	**Z value**	**p value**	**Z value**	**p value**	**Z value**	**p value**
NEN vs. EN	6.90	<0.001	6.33	<0.001	5.03	<0.001	5.57	<0.001
NEN vs. MF	2.77	0.033	2.92	0.021	2.74	0.037	2.93	0.021
NEN vs. CP	3.57	0.002	3.69	0.001	4.75	<0.001	3.25	0.007
EN vs. MF	5.05	<0.001	4.18	<0.001	2.80	0.030	4.04	<0.001
EN vs. CP	4.08	<0.001	3.24	0.007	0.34	1.000	2.84	0.027
MF vs. CP	0.97	1.000	0.95	1.000	2.66	0.040	1.20	1.000
**Kruskal-Wallis H (3, N=70)**	H=53.74, p<0.001		H=43.19, p<0.001		H=31.77, p<0.001		H=34.83, p<0.001	

Analysis: Fig. 3 Antibody Isotypes in sera of *Wuchereria bancrofti* exposed humans to recombinant proteins.

Table S3. IgA Isotype. For each protein, comparison between groups (NEN, EN, MF and CP) were done by Kruskal-Wallis ANOVA and significance of difference in mean ranks between groups was done by Mann-Whitney U test (Z value and p value).

**Comparisons**	**Bm-TPP**		**BmAF-Myo**		**Wol TI IF-1**		***w*Bm-LigA**	
	**Z value**	**p value**	**Z value**	**p value**	**Z value**	**p value**	**Z value**	**p value**
NEN vs. EN	6.72	<0.001	6.54	<0.001	4.95	<0.001	6.10	<0.001
NEN vs. MF	2.98	0.018	2.63	0.031	0.92	1.000	3.18	<0.001
NEN vs. CP	3.63	0.002	3.18	0.009	3.45	0.003	2.52	0.042
EN vs. MF	4.58	<0.001	4.79	<0.001	4.94	<0.001	3.57	0.002
EN vs. CP	3.78	0.001	4.12	<0.001	1.84	0.039	4.63	<0.001
MF vs. CP	0.80	1.000	0.68	1.000	3.10	0.012	1.05	1.000
**Kruskal-Wallis H (3, N=70)**	H= 49.77, p<0.001		H= 48.70, p<0.001		H= 37.37, p<0.001		H= 42.79, p<0.001	

Table S4. IgG1 Isotype. For each protein, comparison between groups (NEN, EN, MF and CP) were done by Kruskal-Wallis ANOVA and significance of difference in mean ranks between groups was done by Mann-Whitney U test (Z value and p value).

**Comparisons**	**Bm-TPP**		**BmAF-Myo**		**Wol TI IF-1**		***w*Bm-LigA**	
	**Z value**	**p value**	**Z value**	**p value**	**Z value**	**p value**	**Z value**	**p value**
NEN vs. EN	5.86	<0.001	5.93	<0.001	6.32	<0.001	5.95	<0.001
NEN vs. MF	3.37	0.005	3.04	0.014	3.12	0.011	3.62	0.002
NEN vs. CP	2.94	0.020	3.77	0.001	3.07	0.013	3.53	0.002
EN vs. MF	3.05	0.014	3.54	0.002	3.92	0.001	2.86	0.025
EN vs. CP	3.58	0.002	2.64	0.046	3.98	<0.001	2.97	0.018
MF vs. CP	0.53	1.000	0.90	1.000	0.06	1.000	0.10	1.000
**Kruskal-Wallis H (3, N=70)**	H= 36.10 , p<0.001		H= 36.77, p<0.001		H= 43.22, p<0.001		H= 35.86, p<0.001	

Table S5. IgG2 Isotype. For each protein, comparison between groups (NEN, EN, MF and CP) were done by Kruskal-Wallis ANOVA and significance of difference in mean ranks between groups was done by Mann-Whitney U test (Z value and p value).

**Comparisons**	**Bm-TPP**		**BmAF-Myo**		**Wol TI IF-1**		***w*Bm-LigA**	
	**Z value**	**p value**	**Z value**	**p value**	**Z value**	**p value**	**Z value**	**p value**
NEN vs. EN	4.45	<0.001	5.74	<0.001	1.94	0.311	6.07	<0.001
NEN vs. MF	3.97	<0.001	3.31	0.006	0.65	1.000	3.02	0.015
NEN vs. CP	2.86	0.025	3.64	0.002	2.00	0.270	3.47	0.003
EN vs. MF	0.58	1.000	2.98	0.018	1.59	0.673	3.74	0.001
EN vs. CP	1.94	0.313	2.58	0.049	0.07	1.000	3.19	0.009
MF vs. CP	1.36	1.000	0.40	1.000	1.66	0.578	0.56	1.000
**Kruskal-Wallis H (3, N=70)**	H= 22.15 , p<0.001		H= 33.48, p<0.001		H= 6.55, p =0.088		H= 38.87, p<0.001	

Table S6. IgG3 Isotype. For each protein, comparison between groups (NEN, EN, MF and CP) were done by Kruskal-Wallis ANOVA and significance of difference in mean ranks between groups was done by Mann-Whitney U test (Z value and p value).

**Comparisons**	**Bm-TPP**		**BmAF-Myo**		**Wol TI IF-1**		***w*Bm-LigA**	
	**Z value**	**p value**	**Z value**	**p value**	**Z value**	**p value**	**Z value**	**p value**
NEN vs. EN	6.58	<0.001	6.12	<0.001	4.45	<0.001	5.52	<0.001
NEN vs. MF	2.71	0.041	3.17	0.009	1.21	1.000	3.81	0.001
NEN vs. CP	3.63	0.002	4.03	<0.001	4.95	<0.001	3.99	<0.001
EN vs. MF	4.75	<0.001	3.60	0.002	3.97	<0.001	3.09	0.022
EN vs. CP	3.62	0.002	2.56	0.043	0.61	1.000	2.86	0.037
MF vs. CP	1.13	1.000	1.04	1.000	4.58	<0.001	0.22	1.000
**Kruskal-Wallis H (3, N=70)**	H= 48.46 , p<0.001		H= 39.12, p<0.001		H= 40.81, p =0.088		H= 30.61, p<0.001	

Table S7. IgG4 Isotype. For each protein, comparison between groups (NEN, EN, MF and CP) were done by Kruskal-Wallis ANOVA and significance of difference in mean ranks between groups was done by Mann-Whitney U test (Z value and p value).

**Comparisons**	**Bm-TPP**		**BmAF-Myo**		**Wol TI IF-1**		***w*Bm-LigA**	
	**Z value**	**p value**	**Z value**	**p value**	**Z value**	**p value**	**Z value**	**p value**
NEN vs. EN	1.53	0.763	2.53	0.068	1.45	0.878	1.97	0.102
NEN vs. MF	2.61	0.054	1.93	0.320	1.83	0.401	1.54	0.542
NEN vs. CP	2.26	0.145	1.00	1.000	1.95	0.304	1.73	0.111
EN vs. MF	1.33	1.000	0.74	1.000	0.47	1.000	0.98	1.000
EN vs. CP	0.89	1.000	1.88	0.357	0.61	1.000	0.24	1.000
MF vs. CP	0.44	1.000	1.15	1.000	0.15	1.000	1.22	1.000
**Kruskal-Wallis H (3, N=70)**	H= 7.69 , p= 0.053		H= 7.87, p= 0.051		H= 4.33, p= 0.228		H= 5.99, p = 0.356	

Table S8. IgE Isotype. For each protein, comparison between groups (NEN, EN, MF and CP) were done by Kruskal-Wallis ANOVA and significance of difference in mean ranks between groups was done by Mann-Whitney U test (Z value and p value).

**Comparisons**	**Bm-TPP**		**BmAF-Myo**		**Wol TI IF-1**		***w*Bm-LigA**	
	**Z value**	**p value**	**Z value**	**p value**	**Z value**	**p value**	**Z value**	**p value**
NEN vs. EN	1.49	0.058	0.33	1.000	2.33	0.118	0.07	1.000
NEN vs. MF	1.68	0.051	2.15	0.188	1.60	0.655	0.26	1.000
NEN vs. CP	1.56	0.053	1.57	0.694	0.33	1.000	0.38	1.000
EN vs. MF	0.38	1.000	1.44	0.741	0.89	1.000	0.23	1.000
EN vs. CP	0.69	1.000	2.33	0.119	2.45	0.085	0.38	1.000
MF vs. CP	1.07	1.00	0.71	1.000	1.56	0.716	0.15	1.000
**Kruskal-Wallis H (3, N=70)**	H= 5.37, p=0.063		H=7.78 , p=0.056		H= 6.75, p =0.051		H= 0.22, p=0.974	

Table S9. IgM Isotype. For each protein, comparison between groups (NEN, EN, MF and CP) were done by Kruskal-Wallis ANOVA and significance of difference in mean ranks between groups was done by Mann-Whitney U test (Z value and p value).

**Comparisons**	**Bm-TPP**		**BmAF-Myo**		**Wol TI IF-1**		***w*Bm-LigA**	
	**Z value**	**p value**	**Z value**	**p value**	**Z value**	**p value**	**Z value**	**p value**
NEN vs. EN	5.50	<0.001	5.88	<0.001	4.40	<0.001	5.51	<0.001
NEN vs. MF	3.27	0.007	3.06	0.013	0.97	1.000	3.43	0.004
NEN vs. CP	3.45	0.003	1.21	1.000	2.09	0.218	2.96	0.018
EN vs. MF	2.73	0.038	3.46	0.003	4.20	<0.001	2.59	0.042
EN vs. CP	2.51	0.043	5.72	<0.001	2.82	0.029	4.11	<0.001
MF vs. CP	0.22	1.000	2.26	0.141	1.38	1.000	1.55	0.721
**Kruskal-Wallis H (3, N=70)**	H= 30.51, p<0.001		H=47.93 , p<0.001		H= 26.25, p<0.001		H= 34.80, p<0.001	

Analysis: Fig. 5 PBMC proliferation of human subjects.

Table S10. For each protein, comparison of stimulation index between groups (EN, MF and CP) were done by Kruskal-Wallis ANOVA and significance of difference in mean ranks between groups was done by Mann-Whitney U test (Z value and p value).

**Comparisons**	**Bm-TPP**		**BmAF-Myo**		**Wol TI IF-1**		***w*Bm-LigA**	
	**Z value**	**p value**	**Z value**	**p value**	**Z value**	**p value**	**Z value**	**p value**
EN vs. MF	2.83	0.014	2.55	0.033	2.90	0.011	2.12	0.010
EN vs. CP	2.47	0.040	2.70	0.026	0.49	1.000	2.07	0.017
MF vs. CP	0.35	1.000	0.85	1.000	2.40	0.049	0.21	1.000
**Kruskal-Wallis H (2, N=15)**	H= 9.50, p=0.009		H=6.72 , p=0.035		H= 9.62, p=0.008		H= 5.46, p=0.065	

Analysis: Fig. 6 Oxidative burst in human PBMCs of bancroftian categories when stimulated with recombinant proteins.

Table S11. For each protein, comparison of ROS generation in terms of mean fluorescence in stimulated PBMC between groups (EN, MF and CP) were done by Kruskal-Wallis ANOVA and significance of difference in mean ranks between groups was done by Mann-Whitney U test (Z value and p value).

**Comparisons**	**Bm-TPP**		**BmAF-Myo**		**Wol TI IF-1**		***w*Bm-LigA**	
	**Z value**	**p value**	**Z value**	**p value**	**Z value**	**p value**	**Z value**	**p value**
EN vs. MF	3.18	0.004	3.11	0.006	2.69	0.022	3.54	0.001
EN vs. CP	2.72	0.010	2.49	0.045	0.07	1.000	2.67	0.023
MF vs. CP	1.06	0.867	0.92	1.000	2.62	0.027	1.77	0.231
**Kruskal-Wallis H (2, N=15)**	H= 10.50, p=0.005		H=10.22 , p=0.006		H= 9.38, p=0.009		H= 12.50, p=0.002	

Analysis of Tables: (Table 2) Results of ADCC assay against B. malayi L3 using human sera

Table S12. Comparison of % killing (Mean ± SD, n=3) between three groups at baseline (no depletion of protein specific antibodies) using Kruskal-Wallis ANOVA

**EN**	**MF**	**CP**	**Kruskal-Wallis H (2, N=9)**	**p value**
84.13 ± 3.61	31.53 ± 1.68	35.00 ± 3.61	6.25	0.044

Table S13. Multiple comparisons of mean ranks of % killing between three groups at baseline by Kruskal-Wallis ANOVA followed by Mann-Whitney U test (Z value and p value).

**Comparisons**	**Z value**	**p value**
EN vs. MF	2.46	0.042
EN vs. CP	1.57	0.035
MF vs. CP	0.89	1.00

Table S14. For each group, comparisons of % killing between different proteins after depletion of protein specific antibodies at baseline using Kruskal-Wallis ANOVA followed by Mann-Whitney U test (Z value and p value).

**Comparisons**	**EN**		**MP**		**CP**	
	**Z value**	**p value**	**Z value**	**p value**	**Z value**	**p value**
Un depleted vs. anti-Bm-TPP depleted	2.83	0.047	2.19	0.285	2.46	0.137
Un depleted vs. anti-BmAF-Myo depleted	1.00	1.000	2.19	0.285	1.05	1.000
Un depleted vs. anti-Wol Tl IF-1 depleted	1.46	1.000	1.78	0.751	2.88	0.040
Un depleted vs. anti-wBm-LigA	2.92	0.035	2.05	0.400	1.83	0.679
**Kruskal-Wallis H (4, N=15)**	H=12.64, p=0.013		H=7.25, p=0.123		H=10.77, p=0.029	
