# Supplementary figures and images for: Humans from Wuchereria bancrofti endemic area elicit substantial immune response to proteins of the filarial parasite Brugia malayi and its endosymbiont Wolbachia
Source: Parasit Vectors. 2017 Jan 24;10:40. doi: 10.1186/s13071-016-1963-x (PMC5259955; doi:10.1186/s13071-016-1963-x)

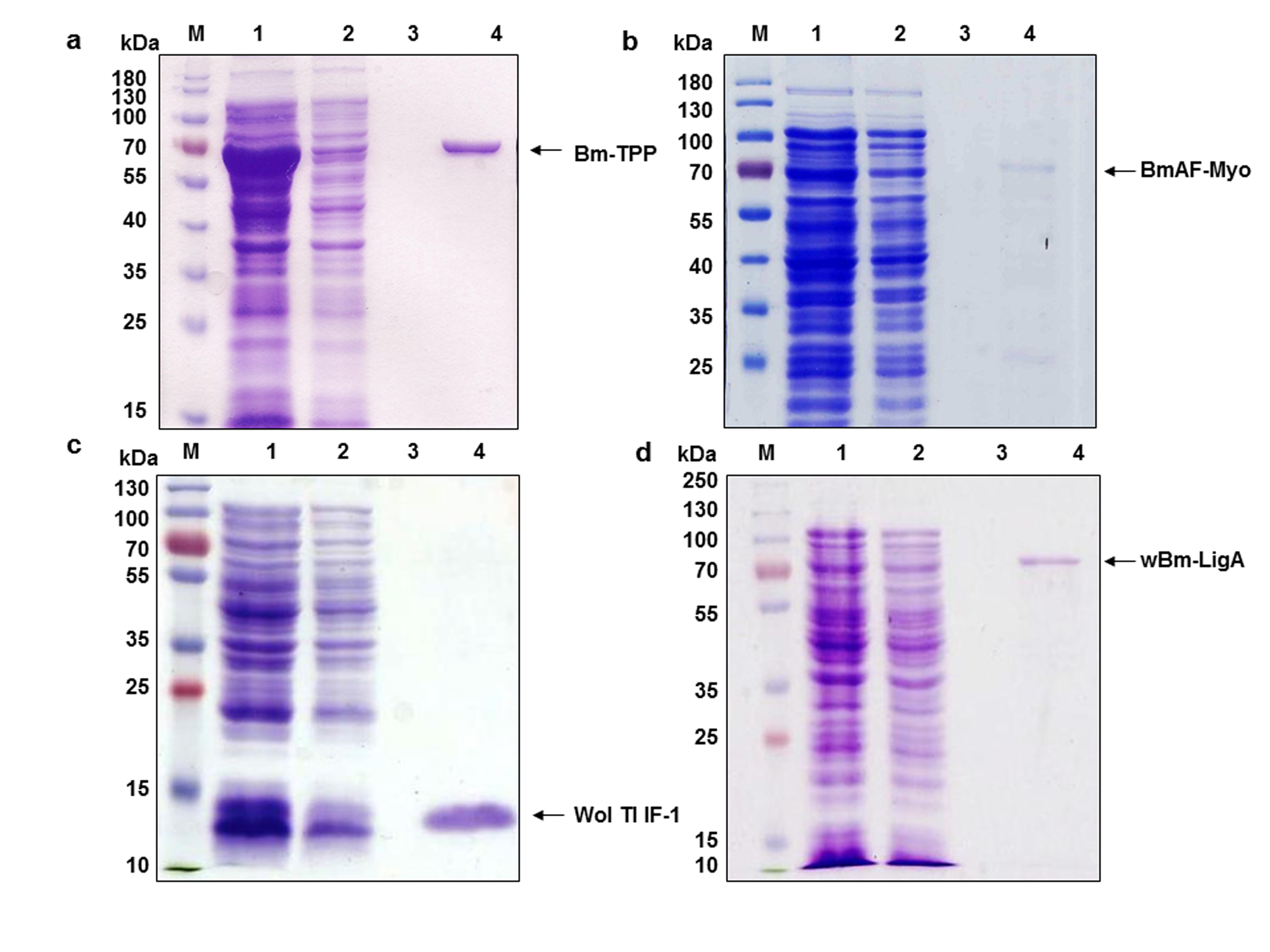

Supplement: Additional file 3: Figure S1. — Overexpression and purification of B. malayi and Wolbachia recombinant proteins. a Coomassie blue stained SDS-polyacrylamide gel. Lane M: standard protein molecular weight marker (Thermoscientific 815-968-0747); Lane 1: soluble E. coli lysates; Lane 2: flow through; Lane 3: wash through prior to elution; Lane 4: purified recombinant Bm-TPP. b Lane M: standard protein molecular weight marker; Lane 1: soluble E. coli lysates after sonication; Lane 2: flow through; Lane 3: wash through; Lane 4: purified recombinant BmAF-Myo. c Lane M: standard protein molecular weight marker; Lane 1: E. coli cell lysates supernatant; Lane 2: flow through; Lane 3: wash through prior to elution; Lane 4: purified eluted recombinant Wol Tl IF-1. d Lane M: standard protein molecular weight marker; Lane 1: soluble cell lysates; Lane 2: flow through; Lane 3:wash through; Lane 4: purifiied eluted wBm-LigA. (TIF 10119 kb) [file 13071_2016_1963_MOESM3_ESM.tif]

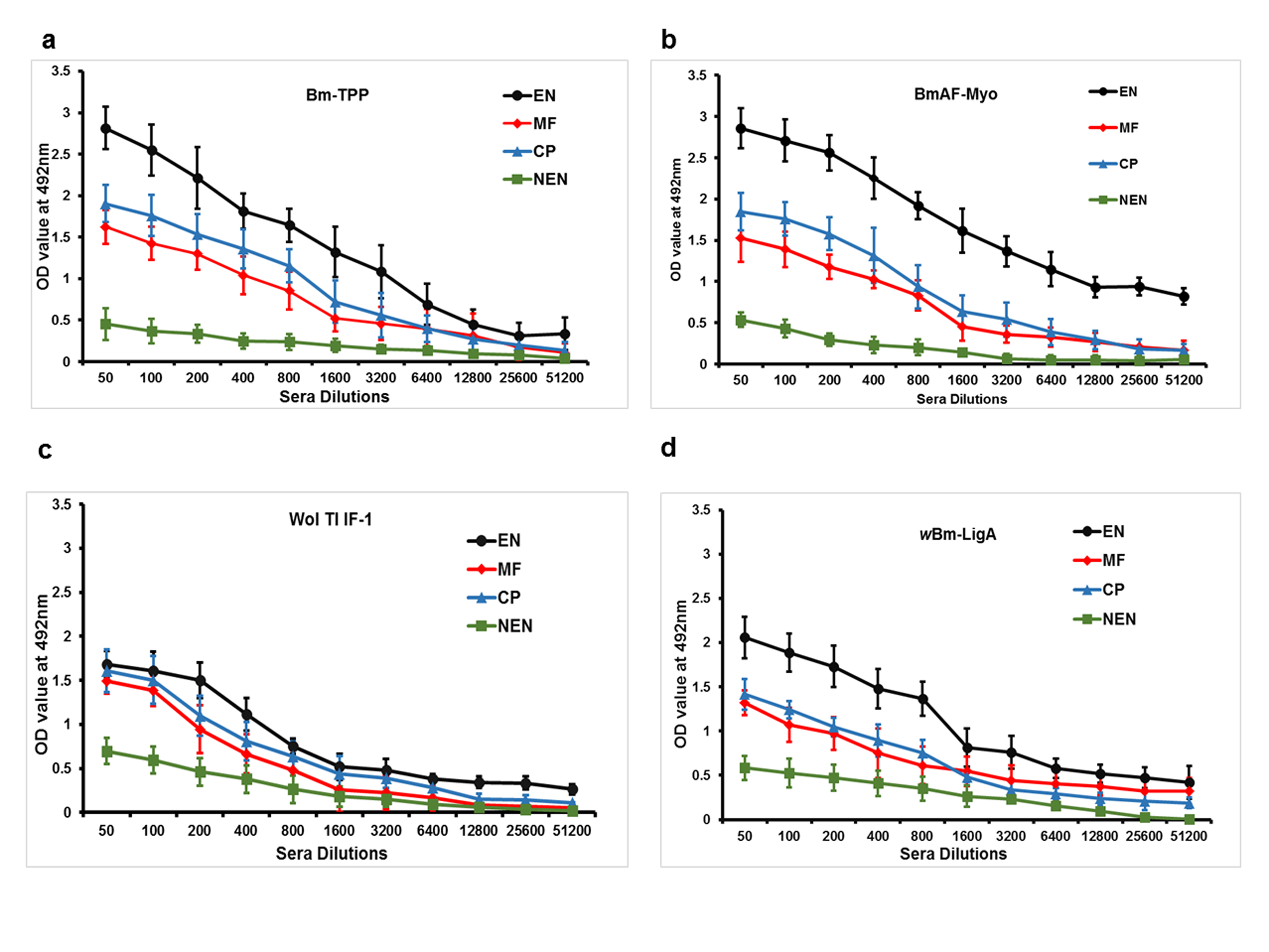

Supplement: Additional file 4: Figure S2. — IgG antibody titer of human bancroftian pateints of different clinical groups and non-filarial individuals. IgG antibody titer values of each category for each protein were evaluated via ELISA by 2-fold serial dilution of pooled human sera that was used as primary antibody. Graph between mean OD values at 492 nm of triplicate wells along with standard errors are plotted on the y-axis against dilution factor along the x-axis for a Bm-TPP; b BmAF-Myo; c Wol Tl IF-1; and d wBm-LigA. (TIF 9547 kb) [file 13071_2016_1963_MOESM4_ESM.tif]
